# Transcriptome Analysis Reveals Complex Defensive Mechanisms in Salt-Tolerant and Salt-Sensitive Shrub Willow Genotypes under Salinity Stress

**DOI:** 10.1155/2020/6870157

**Published:** 2020-07-27

**Authors:** Dezong Sui, Baosong Wang

**Affiliations:** Jiangsu Academy of Forestry, Nanjing, 211153 Jiangsu, China

## Abstract

Salinity stress is one of the most devastating abiotic stresses limiting plant growth and productivity. As a moderately salt-tolerant crop, shrub willow (*Salix* spp.) is widely distributed over the world and can provide multiple bioenergy product and environmental benefits. To delve into the salt tolerance mechanism and screen out salt-tolerant genes, two shrub willow cultivars (a salt-sensitive genotype JW9-6 and a salt-tolerant genotype JW2372) at three time points (0, 2, and 12 h) after NaCl treatments were used for RNA sequencing. A comparative analysis between genotypes and time points showed 1,706 differentially expressed genes (DEGs), of which 1,029 and 431 DEGs were only found in the JW9-6 and JW2372, respectively. Gene Ontology (GO) and MapMan annotations suggested that many DEGs were involved in various defense-related biological pathways, including cell wall integrity, hormone signaling, antioxidant system, heat shock proteins, and transcription factors. Compared to JW9-6, JW2372 contained more DEGs involved in the maintenance of the cell wall integrity, ABA, and ethylene signal transduction pathways. In addition, more DEGs encoding heat shock proteins were found in JW2372. Instead, transcription factors including ERF, MYB, NAC, and WRKY were found to be more differentially expressed in JW9-6 under salinity stress. Furthermore, expressions of nine randomly selected DEGs were verified by qRT-PCR analysis. This study contributes in new perspicacity into underlying the salt tolerance mechanism of a shrub willow at the transcriptome level and also provides numerous salt-tolerant genes for further genetic engineering and breeding purposes in the future.

## 1. Introduction

Abiotic stresses such as drought, cold, heat, and salinity largely threaten agricultural productivity worldwide [1]. Among these environmental stress factors, salinity is a major abiotic stress that results in reduced plant growth and a significant reduction in productivity. Globally, an estimate of 20% of the world's cultivated lands and nearly 33% of the world's irrigated lands are affected by salinity, and this area is predicted to be larger by 2,050 [2]. A high salt concentration usually limits plant growth via several distinct processes including increasing soil osmotic pressure, interference with plant nutrition, and ion imbalances. Moreover, salinity could result in metabolic dysfunctions, photosynthesis inhibition, and cellular structure damage [3]. Therefore, understanding the physiological and genetic mechanisms of salinity stress responses in plants can provide valuable information for developing salt-tolerant plants with additional salt-responding traits.

Through evolution, plants have evolved various physiological and biochemical mechanisms to withstand salinity stress and then respond with a series of complex processes at the molecular, metabolic, and cellular levels. In recent years, research into salt tolerance has been carried out in many plants, such as tomato [4], rice [5], cotton [6], and maize [7]. Generally, with increasing investigation into plant salt tolerance, plants adapt to salinity stress via the ways including osmotic regulation, ion uptake and transport, antioxidant metabolism, hormone metabolism, and stress signaling [8–10]. Salinity can stimulate the production of reactive oxygen species (ROS) in plants, which results in oxidative stress. In maintenance of osmotic homeostasis, some plants accumulate osmoprotectants, and some plants use antioxidant mechanisms to eliminate ROS [11]. The ROS-scavenging systems in plants contain various antioxidant enzymes, such as catalase, superoxide dismutase, glutathione reductase, and ascorbate peroxidase [12]. Moreover, numerous signaling sensors such as various transcription factors are integral in linking salt-sensory pathways, which enable plants to nurture and transcend the adverse conditions [13]. Some transcription factor family genes including WRKY, ERF, MYB, and bHLH families, have been reported to be differentially expressed in response to external salinity [14]. These transcription factors regulate the expression levels of genes that ultimately affect the level of plant salt tolerance. Plant hormones including abscisic acid (ABA), ethylene, auxin, and salicylic acid (SA) have been reported to participate in the adaption of salinity stress. Some genes involved in the hormone signaling pathway have been investigated to play an important role in the response to salinity stress. For example, overexpressing of *Gossypium hirsutum* sucrose nonfermenting 1-related protein kinase 2 (GhSnRK2), which acts as a positive regulator in the ABA signaling pathway, exhibited increased tolerance to salinity stress [15].

Shrub willow (*Salix* spp.) is a short-rotation woody crop that provides multiple bioenergy products and environmental benefits. It also supplies attractive biomass because it is high yielding, fast growing, and requires few inputs. One of the most noteworthy advantages is that shrub willow can grow successfully in a range of drainage conditions, from poorly to well-drained fallow land, and can also enhance soil properties and microbial diversity. Over hundreds of shrub willow genotypes with diverse characteristics are found, and many of these genotypes are salt-tolerance to some extent, including *Salix mongolica*, *S*. *triandra Thrnb*, and *S*. *integra*. Therefore, screening and breeding new genotypes with high salt-tolerance have become a challenging task. Previously, our group successfully established an evaluation system to evaluate salt tolerance for shrub willow genotypes based on multiple factors including plant height, relative water content, and transpiration [16, 17]. Based on this strategy, a salt-tolerant genotype JW2372 and a salt-sensitive genotype JW9-6 were identified. Currently, there are few studies on the physiological and biochemical processes of shrub willow under salinity stress. Therefore, the salt-tolerant genotype JW2372 and the salt-sensitive genotype JW9-6 supply a good opportunity to investigate the physiological and genetic mechanisms of salinity stress responses in shrub willow.

In recent years, next-generation sequencing technology including high-throughput RNA-Seq technology has been extensively applied to unveil the diversity of salinity stress on a transcriptome-wide scale in plants. Comparative transcriptional analysis could identify and characterize the differentially expressed genes involved in salinity stress responses. There have been reports aimed at investigating the salt tolerance mechanisms in many model and nonmodel plants, such as rice [18], peanut [19], soybean [20], and cotton [6]. In asparagus bean, a salt-sensitive genotype and a salt-tolerant genotype under salinity stress vs. the control were used to identify salt-stress-induced genes by RNA-Seq sequencing, and diverse transcription factors were found to be involved in the plant salt tolerance process [21]. Comparison of the transcriptome changes between salt-tolerant and salt-sensitive upland cotton cultivars in response to NaCl treatments revealed that signal transduction, transcriptional regulation, and secondary metabolism showed significant differences in two varieties [6]. However, transcriptome sequencing studies of shrub willow under salinity stress are rare, and the molecular basis of salinity stress tolerance in shrub willow remains largely unknown.

In the present study, two shrub willow cultivars (one salt-sensitive genotype JW9-6 and one salt-tolerant genotype JW2372) were treated with NaCl at 0 h, 2 h, and 12 h, followed by RNA-Seq sequencing. The aim was to investigate whole-transcriptome expression profiles of genes in shrub willow under salinity conditions at early time points. The analysis of differentially expressed genes related to salinity stress would help us in understanding the functional and molecular mechanisms underlying salt tolerance in shrub willow.

## 2. Materials and Methods

### 2.1. Plant Material and Salinity Stress Treatment

Two shrub willow cultures, a salt-sensitive genotype JW9-6 and a salt-tolerant genotype JW2372, identified by our group were employed in this study [16, 17]. The two genotypes were cultivated in the nutrient solution containing 1/4 Hoagland, which was replaced with fresh solution every seven days. The salinity stress treatment was conducted according to previously established methods with some modifications [22]. The seedlings were grown in a growth chamber under 22-28°C. Six-week-old seedlings were planted in each ampulla (300 mL) with 1/4 Hoagland nutrient solution including 3% NaCl, and the controls were planted in the nutrient solution containing 1/4 Hoagland. Seedling samples which were taken at different salinity stress time points (2 h and 12 h) were immediately frozen in liquid nitrogen and then stored at -70°C.

### 2.2. RNA Extraction and Sequencing

Total RNAs were extracted from these samples using the RNA simple Total RNA Kit (Tiangen, China) according to the manufacturer's instructions and were then treated with DNase I to remove potential genomic DNA contamination. After that, the RNAs were examined by gel electrophoresis and quantified with NanoDrop. Integrity of the quantified RNA samples was analyzed using an Agilent 2100 Bioanalyzer (Agilent Technologies, USA). The cDNA libraries were constructed using TruSeq RNA Sample Preparation V2 (Illumina, USA) according to the manufacturer's instructions. After quality control to detect fragment size and concentration, the libraries were sequenced using the Illumina HiSeq 2500 system. All sequence data were uploaded to the NCBI SRA database with the SRA accession number PRJNA639011.

### 2.3. Transcriptome Analysis

The *Salix purpurea* V1.0 reference genome was retrieved from JGI website (https://phytozome.jgi.doe.gov/pz/portal.html#!info?alias=Org_Spurpurea). After removing reads of low quality, the produced obtained clean reads were aligned to the *S*. *purpurea* genome using TopHat with default parameters [23]. Only the uniquely mapped reads were retained for subsequent processing. The mapped reads were quantified using Cufflinks software [24], and the expression level of each gene was calculated by normalizing to the fragment per kilobase of exon per million mapped reads (FPKM) value. To identify differentially expressed genes between samples, the Cuffdiff algorithm embedded in the Cufflinks software was applied. Genes with two fold-change and a *q* value of less than 0.05 were considered differentially expressed between two samples. AgriGO was used to perform the gene ontology (GO) analysis for functional categorization of differentially expressed genes [25]. MapMan ontology tool was used to obtain an overview of differentially expressed genes involved in metabolic pathways, in which a plant-specific ontology classifies genes into well-defined hierarchical categories and were denominated BINs [26].

### 2.4. qRT-PCR Validation of Differentially Expressed Genes

To validate the identified DEGs, a total of nine genes deriving from various pathways were randomly selected for qRT-PCR analysis. The primers for these genes were designed with Primer5 software (Table S1). The total RNA was extracted using the same method described above. The RNA was then reverse-transcribed using the PrimeScript First-strand cDNA Synthesis Kit (TaKaRa, Japan) according to the manufacturer's instructions. The qRT-PCR reactions were carried out on an ABI PRISM 7500 Real-Time PCR System (Applied Biosystems) under the following conditions: 95°C for 5 min, 10 s at 95°C for 10 s, and 60°C for 34 s for 40 cycles to calculate cycle threshold values, followed by a dissociation program of 95°C for 15 s, 60°C for 1 min, and 95°C for 15 s to obtain melt curves. With the actin gene as an endogenous control, the relative expression level of each tested gene was normalized to the expression level of reference gene calculated using the 2^-*ΔΔ*Ct^ method [27]. Significant statistical differences between the salt treatment and the control group were analyzed using Student's *t*-test (^∗^*P* < 0.05, ^∗∗^*P* < 0.01).

## 3. Results

### 3.1. Summary of RNA-Seq Data Sets

In order to explore the transcriptome change and salt-tolerant genes of shrub willow at early time points, the salt-sensitive genotype JW9-6 and the salt-tolerant genotype JW2372 previously identified by our group were used [16, 17]. Based on the established evaluation system, JW2372 exhibited significantly higher biomass (fresh weight), stem length, ground diameter, and survival rate than JW9-6 after NaCl treatment [16, 17]. In addition, some physiological parameters including proline content, soluble protein content, superoxide dismutase (SOD) activity, peroxidase (POD) activity, catalase (CAT) activity, and photosynthetic rate, exhibited obvious changes at 2 and 12 h after NaCl treatment [28]. Therefore, in this study, a total of 18 cDNA libraries were constructed for the two shrub willow genotypes treated with NaCl for 0, 2, and 12 h and then sequenced using the Illumina deep sequencing platform. Three biological replicates were applied for each sample. The produced raw reads were processed to remove adapter sequences and low-quality sequences, resulting in an average of 28.4 million clean reads for each sample (Table S2). These clean reads were then mapped onto the *Salix purpurea* genome using TopHat software, and the mapped reads ranged from 83.0% to 87.4% for each sample (Table S2), respectively. To obtain the expression value of each gene in *S*. *purpurea*, we calculated the fragment per kilobase of exon per million mapped reads (FPKM) for each gene. Of the 37,865 annotated genes in the *S*. *purpurea* genome, 27,265 (72.0%) genes were detected in at least one sample, and 20,029 (52.9%) genes were expressed in each sample.

### 3.2. Identification of Differentially Expressed Genes

**Figure 1:**
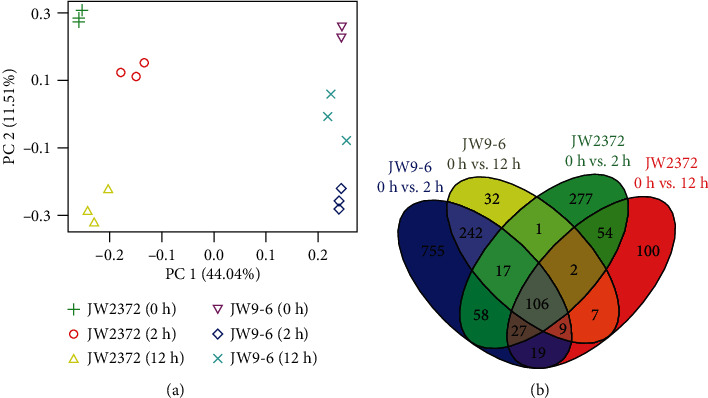
Venn diagram analysis of identified DEGs under salinity stress in shrub willow. (a) Principal component analysis of all samples. (b) Venn diagrams showing unique and shared DEGs between time points under salinity stress.

Based on the principal component analysis of all samples, the transcriptional response observed in shrub willow exposed to salinity stress was due to NaCl treatment, and the salt-sensitive genotype JW9-6 and the salt-tolerant genotype JW2372 exhibited different levels of gene expression (Figure 1(a)). Moreover, the three biological replicates of each library had the similar level of gene expression (Figure 1(a)), indicating that the current RNA-Seq data were reliable for subsequent analysis. To further identification of differentially expressed genes (DEGs) in response to salinity stress, two fold-change and a *q* value of less than 0.05 were set as the cutoff threshold. The numbers of DEGs at different time points were summarized in Table 1 and Figure 1(b). A comparative analysis between genotypes and time points showed a total of 1,706 DEGs, of which 1,029 and 431 DEGs were only found in the JW9-6 and JW2372, respectively. After the salt-sensitive genotype JW9-6 was exposed to salinity stress, 1,233 DEGs were identified at 2 h while 416 DEGs were found at 12 h. The result showed that upregulated genes were significantly larger than downregulated genes at 2 h and 12 h, and almost three times the number of genes was found to be differentially expressed at 2 h than that at 12 h (Table 1 and Figure 1(b)). Similarly, for the salt-tolerant genotype JW2372, a total of 542 DEGs were found at 2 h, which is larger than the identified 324 DEGs at 12 h. The comparison also showed that JW9-6 had more DEGs than JW2372 at each time point. Furthermore, a total of 106 DEGs were found in the overlapping region of these four comparisons (Figure 1(b)), which indicated that these genes were differentially expressed at each time point regardless of genotype when subjected to salinity stress. There were 242 DEGs found at both 2 h and 12 h in JW9-6, while 755 and 32 genes were only differentially expressed at 2 h and 12 h, respectively. Corresponding to JW2372, 54 genes were differentially expressed at both 2 h and 12 h, while 277 and 100 DEGs were found only at 2 h and 12 h, respectively (Figure 1(b)). These results revealed that the salt-sensitive genotype JW9-6 triggered more DEGs in response to salinity stress.

### 3.3. Functional Classification of Differentially Expressed Genes

**Figure 2:**
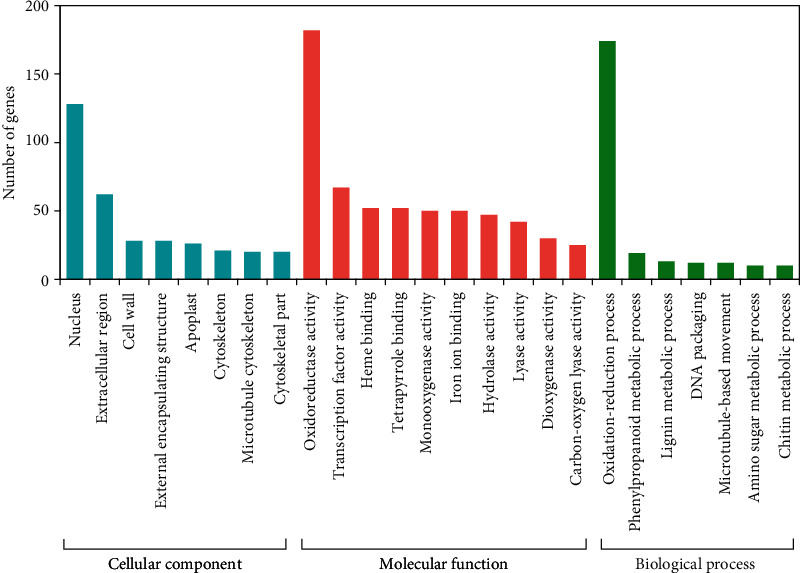
Gene ontology (GO) classifications of DEGs. The DEGs are classified into three main categories: biological processes, cellular components, and molecular functions. The *X*-axis indicates the categories, and the *Y*-axis represents the number of DEGs in a category.

To investigate possible biological functions of these 1,706 DEGs identified in the two genotypes, they were functionally annotated based on gene ontology (GO) enrichment analysis. A total of 1,051 DEGs were assigned to at least one GO term and categorized into >50 categories (Table S3). In the molecular function group, GO terms such as “catalytic activity” (GO:0003824), “oxidoreductase activity” (GO:0016491), “transcription factor activity” (GO:0003700), and “iron ion binding” (GO:0005509) were significantly enriched (Figure 2), indicating that genes acting in these processes might play crucial roles in the response to NaCl treatment. In addition, “oxidation-reduction process” (GO:0055114), “phenylproganoid metabolic process” (GO:0009698), and “lignin metabolic process” (GO:0009808) were the most represented in the category of biological process group. Furthermore, “nucleus” (GO:0005634) and “extracellular region” (GO:0005576) were the main categories in the cellular component group (Figure 2).

### 3.4. Maintenance of Cell Wall Integrity

Plant cell wall, as the first barrier to environmental stress, should respond fast by modulating its composition and structure. Thus, cell wall integrity contributes greatly under abiotic stresses [29]. A total of 40 DEGs encoding cell wall-related proteins were identified under salinity stress (Table S4). Four genes encoding galacturonosyltransferase, galactoside 2-alpha-L-fucosyltransferase, and cellulose synthase were significantly upregulated in JW2372 rather than JW9-6. Expansins are cell wall proteins that improve plant stress tolerance [30–32]. Consistent with this phenomenon, two expansins were uniquely upregulated at 12 h in JW2372, whereas another two expansins were repressed by salinity stress in JW9-6. Moreover, five genes encoding xyloglucan endotransglucosylase were also uniquely induced in JW2372, suggesting that they participated in cell wall modification. Notably, six and four genes involved in lignin biosynthesis were induced in JW9-6 and JW2372, which could contribute to increased lignin accumulation that promote cell wall rigidity during salinity stress [32–34]. Overall, these results suggested that JW2372 could maintain better cell wall plasticity than JW9-6 under salinity stress.

### 3.5. Plant Hormone Signal Transduction Changes

**Figure 3:**
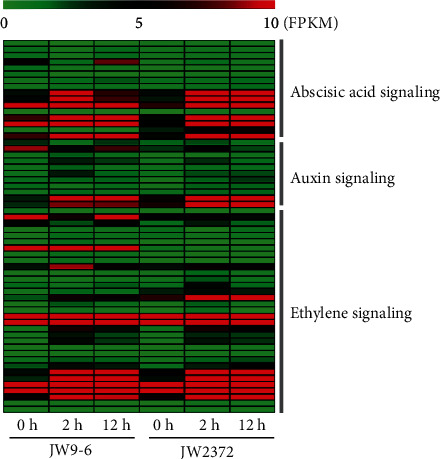
The heat map of DEGs related to plant hormone signaling pathways. Only DEGs related to ABA, auxin, and ethylene signaling pathways are shown. Each line refers to one gene. The color bar represents the FPKM value.

It is known that plant hormones play a vital role in the plant's ability to adapt to changing environments, including salinity stress [35]. In this study, various DEGs encoding hormone signaling were mainly involved in ABA, auxin, and ethylene synthesis and signal transduction pathways (Figure 3 and Table S5). The ABA signaling pathway plays a protective role in plants against salinity stress. In the ABA synthesis process, two genes encoding 9-cis-epoxycarotenoid dioxygenase were induced in JW9-6 and JW2372 under salinity stress, while three UDP-glycosyltransferase were suppressed in JW9-6. Another two genes encoding protein phosphatase 2C (PP2C) were only upregulated in JW2372 at 12 h. Many genes involved in ethylene biosynthesis and signal transduction were induced or repressed in response to salinity stress, including homologs of 1-aminocyclopropane-1-carboxylate oxidase, ERF transcription factor, and ethylene-responsive nuclear protein (Table S5). Notably, three genes encoding ethylene-responsive nuclear protein, and two genes encoding 1-aminocyclopropane-1-carboxylate oxidase, were specifically induced in JW2372. Moreover, homologs of genes which were associated with auxin signal transduction pathway were induced or repressed under NaCl treatment. Several genes encoding IAA-amido synthase and SAUR-like auxin-responsive protein were specifically induced in both of JW9-6 and JW2372 at 2 h (Table S5). Two genes encoding IAA amino acid conjugate hydrolase were only repressed in JW9-6 at 2 h. Together, these results demonstrated that hormones form a complex regulatory network related to the stress response to cope with salinity stress.

### 3.6. Increased Antioxidant Systems

High salinity usually stimulates the production of reactive oxygen species (ROS), which negatively affects plant cellular processes. In response, plants accumulate various antioxidant enzymes to quench free radicals. In the current study, 23 DEGs were identified as enzymes in the ROS detoxification system. These DEGs were mainly composed of ROS enzymes encoding peroxidase (POD) and glutathione S-transferase (GST) (Table S6). A total of five POD were upregulated at 2 h or 12 h for the salt-sensitive genotype JW9-6, while only 2 PODs were induced at 2 h for the salt-tolerant genotype JW2372 (Table S6). Moreover, 12 GSTs were induced in JW9-6 under salinity stress, while only 3 GSTs were upregulated in JW2372. Plant GSTs are known to detoxify xenobiotics and hydroperoxides and function in abiotic stress responses. In both of JW9-6 and JW2372, more PODs and GSTs were induced at 2 h compared to 12 h after NaCl treatment (Table S6). These findings suggested a common response of antioxidant enzymes to detoxify ROS effects.

### 3.7. Activated Heat Shock Proteins

**Figure 4:**
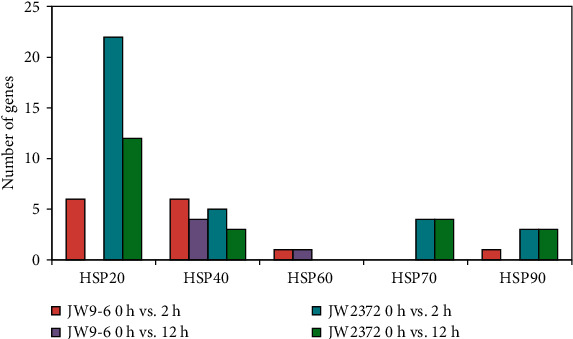
Distribution of differentially expressed heat shock proteins.

Heat shock proteins (HSPs) are the molecular chaperones that act as stress-responsive proteins, thus protecting plants from stress damage. Overall, 39 genes encoding HSPs were differentially expressed, and all of them were upregulated under salinity stress in this study (Table S7). These HSPs were composed of 22 HSP20s, 9 HSP40s, 1 HSP60, 4 HSP70s, and 3 HSP90s (Figure 4), suggesting that low molecular weight HSPs played a more important role than the high molecular weight HSPs in response to salinity stress. Furthermore, a total of 34 and 22 HSPs were induced at 2 h and 12 h in JW2372, while only 13 and 5 HSPs were upregulated in JW9-6 (Figure 4), indicating that HSPs played a more central role in the salt-tolerant genotype JW2372. Notably, more upregulated HSPs were found at 2 h compared to that at 12 h, suggesting that HSPs were rapidly activated in the very early stage of salinity stress treatment.

### 3.8. Induced Transcription Factors

**Figure 5:**
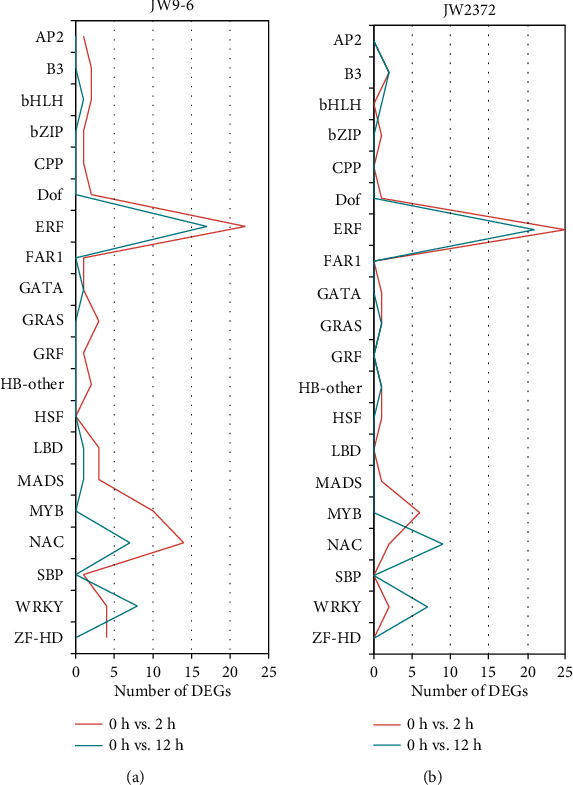
Prevalence of differentially expressed transcription factor families after salinity stress. (a) DEGs encoding transcription factors in JW9-6. (b) DEGs encoding transcription factors in JW2372. Only transcription factor families with more than one DEG between any comparison are shown.

In recent years, several transcription factors (e.g., ERF, MYB, NAC, and WRKY) have been verified to function as molecular switches to regulate the expression of stress-related genes. In this study, a total of 118 transcription factors (TFs) were differentially expressed under salinity stress, and they were classified into 20 different families based on family assignment rules of PlantTFDB (Table S8). Notably, over two thirds of differentially expressed TFs belonged to ERF, MYB, NAC, and WRKY families (Figure 5). In the salt-sensitive genotype JW9-6, 69 TFs were induced at 2 h and 43 TFs were upregulated at 12 h (Figure 5(a)), indicating that more TFs were activated in the very early stage of salinity stress treatment. These activated TFs mainly consisted of ERF (24.4%), NAC (21.8%), WRKY (11.5%), MYB (7.7%), and ZF-HD (5.1%). However, only 43 and 40 TFs were induced in the salt-tolerant genotype JW2372 at 2 h and 12 h (Figure 5(b)), respectively, and these activated TFs mostly belonged to ERF (42.4%), NAC (15.3%), WRKY (15.3%), and MYB (8.5%). Interestingly, 17 ERF and 1 NAC TFs were induced at each time point regardless of genotype when subjected to salinity stress. Overall, in comparison to JW2372, JW9-6 showed larger number of activated TFs, suggesting that the response to salinity stress was more rapid in the salt-sensitive genotype JW9-6.

### 3.9. Validation of Salinity Stress-Induced DEGs by qRT-PCR

**Figure 6:**
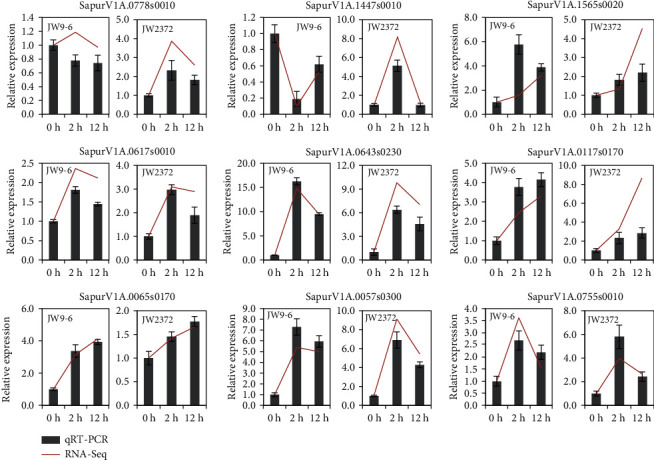
Validation of DEGs by qRT-PCR. The relative expression levels of ten randomly selected DEGs are verified by qRT-PCR. The expression levels at 0 h are arbitrarily set to 1. Error bars represent the SD for three biological replicates.

In order to validate the DEGs identified by RNA-Seq analysis, nine genes induced after salinity stress were randomly selected to analyze their expression by qRT-PCR. The selected genes included two ERF transcription factors, two HSPs, one POD, one acyl-transferase, one cytochrome P450, one protein phosphatase 2C, and one nine-cis-epoxycarotenoid dioxygenase. The results showed that all the nine genes had similar expression patterns in the qRT-PCR and RNA-Seq data (Figure 6), which highlighted the reproducibility and accuracy of the RNA-Seq analysis.

## 4. Discussion

**Figure 7:**
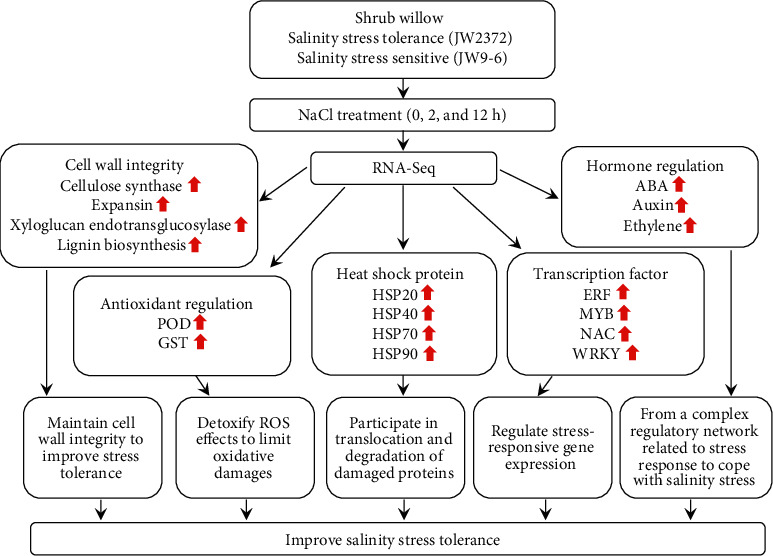
A schematic complex regulatory mechanism of salinity stress tolerance in shrub willow. Upon exposure to salinity stress, genes involved in cell wall integrity, antioxidant regulation, heat shock protein, hormone regulation, and transcription factor were upregulated in shrub willow. Red arrows represent upregulated genes. POD: peroxidase; GST: glutathione S-transferase; ABA: abscisic acid; HSP: heat shock protein.

In the present study, RNA-Seq sequencing of two shrub willow cultivars (one salt-sensitive genotype JW9-6 and one salt-tolerant genotype JW2372) were performed to investigate genes involved in salt tolerance mechanisms. A total of 1,706 DEGs were identified in JW9-6 and JW2372 seedlings between control and salt-treated conditions. Based on GO analysis, the dominant GO terms of the DEGs were comprised of oxidoreductase activity, transcription factor activity, and iron ion binding process. Further analysis revealed that genes related to cell wall integrity, hormone signaling, antioxidant system, heat shock proteins, and transcription factors, were differentially expressed (Figure 7). Notably, compared to JW2372, JW9-6 contained more DEGs at 2 h and 12 h. This phenomenon is consistent with that found in asparagus bean that the salt-tolerant cultivar triggered much more DEGs both in leaves and in roots [21].

Salinity stress affects the large-scale metabolic activities that lead to excessive ROS accumulation. Thus, the balance between ROS production and quenching is critical under salinity stress. Plants have evolved a complex antioxidative defense system to limit the oxidative damage, which mainly comprised of enzymatic antioxidant (SOD, CAT, POD, and GST) [36]. In the present study, the antioxidative defense system was activated in salt-treated shrub willow based on observation of differentially expressed PDD and GST genes, although SOD and CAT genes were not differentially expressed. Similarly, overexpression of the GST gene in tobacco enhanced the tolerance to salt stress [37]. In addition, POD and GST genes were observed preferentially to be induced at 2 h compared to 12 h after salt treatment in shrub willow, suggesting that these antioxidant enzymes were activated rapidly at the very early stage.

HSPs are the molecular chaperones known to participate in the translocation and degradation of damaged proteins under abiotic stresses. In this study, a total of 38 HSPs were upregulated, while no downregulated HSP was found under salinity stress. Further analysis showed that low molecular weight HSPs played a more important role than the high molecular weight HSPs. In addition, more induced HSPs were identified in the salt-tolerant genotype JW2372, suggesting that HSPs played a more central role in salt tolerance process. Some HSPs were also found to be activated in tartary buckwheat and grapevine, indicating their regulatory role in various signaling-related pathways [38, 39].

Transcription factors have been reported to participate in regulating the stress-responsive gene expression in many plants responding to abiotic stress. A number of MYB genes have been studied to induce plant responses to salinity stress acclimation. In rice, the expression of a MYB gene (*OsMYB2*) was upregulated by salinity stress, and the *OsMYB2*-overexpressing plants were more tolerant to salt than wild-type plants [40]. In tobacco, overexpression of the MYB gene (TaODORANT1) induced the expression of some ROS- and stress-related genes in response to both drought and salinity stresses, thus positively regulating plant tolerance to drought and salinity stresses [41]. In our study, 10 and 6 MYB genes were differentially expressed in JW9-6 and JW2372 under salinity stress, and all of these DEGs were upregulated, which was consistent with that observed in other plants. The ERF genes are also linked to salinity stress responses. In cotton, the transcripts of *GhERF4* accumulate highly when plants are treated with salt, cold, and drought stresses [42]. Our study showed that 24.4% and 42.4% of activated TFs belonged to ERF genes in JW9-6 and JW2372, respectively, which indicated the complexity of the ERF regulatory mechanism. In addition, WRKY gene family is regarded as the essential TFs involved in salinity stress responses, such as *GhWRKY39* in cotton [43] and *WRKY33* in *Arabidopsis* [44]. In the current study, 9 WRKY TFs were upregulated, and more induced DEGs were identified at 12 h than 2 h, which suggested that WRKY TFs were not rapidly at the very early stage in shrub willow.

## 5. Conclusion

In conclusion, this study provides a comprehensive overview of the transcriptome characteristics to understand the complex molecular mechanisms involved in response to salinity stress in shrub willow. Moreover, candidate genes related to salt tolerance identified here can be applied in further research to improve the salt tolerance of shrub willow and other plants.

## Figures and Tables

**Table 1:** Number of DEGs at different salinity stress time points in the two genotypes.

	Upregulated	Downregulated	Total DEGs
JW9-6_0 h versus JW9-6_2 h	1,024	209	1,233
JW9-6_0 h versus JW9-6_12 h	404	12	416
JW2372_0 h versus JW2372_2 h	525	17	542
JW2372_0 h versus JW2372_12 h	289	35	324

## Data Availability

The datasets used and analyzed during the current study are available from the corresponding author upon reasonable request.

## Supplementary Materials

Supplementary 1 Table S1: designed primers for qRT-PCR validation in this study.

Supplementary 2 Table S2: summary of RNA-Seq data and mapping results.

Supplementary 3 Table S3: list of enriched GO categories in the DEGs.

Supplementary 4 Table S4: DEGs encoding cell wall-related proteins under salinity stress.

Supplementary 5 Table S5: DEGs involved in hormone metabolism under salinity stress.

Supplementary 6 Table S6: DEGs involved in antioxidant system under salinity stress.

Supplementary 7 Table S7: DEGs encoding heat shock proteins under salinity stress.

Supplementary 8 Table S8: DEGs encoding transcription factors under salinity stress.
