# Supplementary material for: Transcriptome Analysis Reveals Complex Defensive Mechanisms in Salt-Tolerant and Salt-Sensitive Shrub Willow Genotypes under Salinity Stress
Source: Int J Genomics. 2020 Jul 27;2020:6870157. doi: 10.1155/2020/6870157 (PMC7407064; doi:10.1155/2020/6870157)
Supplement: Supplementary 1 — Table S1: designed primers for qRT-PCR validation in this study. [file 6870157.f1.doc]

Table S1. Designed primers for qRT-PCR validation in this study.

| Primer name | Primer sequence | Purpose |
| --- | --- | --- |
| S0778RT-F | AGCGGCAAAACCGCGGAGAT | qRT-PCR analysis of SapurV1A.0778s0010 |
| S0778RT-R | CTCCAGCCGGTGGCGACAAT |
| S1447RT-F | TGGTCCCCTCATGTCCCTCCG | qRT-PCR analysis of SapurV1A.1447s0010 |
| S1447RT-R | CACGTGAGGCACACACCGGC |
| S1565RT-F | ACAGCGGTTGTTGCGGTGGT | qRT-PCR analysis of SapurV1A.1565s0020 |
| S1565RT-R | CTCATCAGGCCTGTCGGGCT |
| S0617RT-F | CGCCCGATGTTGAAATCCCGCT | qRT-PCR analysis of SapurV1A.0617s0010 |
| S0617RT-R | TCACCGGAGATCCTCCCCGA |
| S0643RT-F | AGGCAACGGCCATGGGGAAA | qRT-PCR analysis of SapurV1A.0643s0230 |
| S0643RT-R | TGGCTGCCTTGTCATAAGCCCT |
| S0117RT-F | ACCACAGCCGAGCGTTGACT | qRT-PCR analysis of SapurV1A.0117s0170 |
| S0117RT-R | TGGACACAACATCAGGGTGGCA |
| S0065RT-F | CCTGCCCCGGTGTTGTGTCC | qRT-PCR analysis of SapurV1A.0065s0170 |
| S0065RT-R | CGCTAACACTTCCTGCTGTGGC |
| S0057RT-F | CGGGCGGTCACTCAGCCATC | qRT-PCR analysis of SapurV1A.0057s0300 |
| S0057RT-R | TGCCCAGCCCCGCATTCAGA |
| S0755RT-F | TGCCAGGACTGTCCAAGGAAGA | qRT-PCR analysis of SapurV1A.0755s0010 |
| S0755RT-R | CCTGCAGCCTAAGCCGGGTA |
