# Supplementary material for: Transcriptome Analysis Reveals Complex Defensive Mechanisms in Salt-Tolerant and Salt-Sensitive Shrub Willow Genotypes under Salinity Stress
Source: Int J Genomics. 2020 Jul 27;2020:6870157. doi: 10.1155/2020/6870157 (PMC7407064; doi:10.1155/2020/6870157)
Supplement: Supplementary 2 — Table S2: summary of RNA-Seq data and mapping results. [file 6870157.f2.doc]

**Table S2**. Summary of RNA-Seq data and mapping results.

| Samples | Replicate | Read number | Base Number | Read aligned |
| --- | --- | --- | --- | --- |
| JW9-6_0 h | Replicate 1 | 50,185,088 | 7,527,763,200 | 87.41% |
| JW9-6_0 h | Replicate 2 | 49,961,698 | 7,494,254,700 | 87.43% |
| JW9-6_0 h | Replicate 3 | 55,802,028 | 8,370,304,200 | 87.35% |
| JW9-6_2 h | Replicate 1 | 49,804,424 | 7,470,663,600 | 87.31% |
| JW9-6_2 h | Replicate 2 | 51,550,938 | 7,732,640,700 | 87.25% |
| JW9-6_2 h | Replicate 3 | 60,044,828 | 9,006,724,200 | 87.33% |
| JW9-6_12 h | Replicate 1 | 52,481,472 | 7,872,220,800 | 86.45% |
| JW9-6_12 h | Replicate 2 | 70,004,394 | 10,500,659,100 | 86.69% |
| JW9-6_12 h | Replicate 3 | 55,516,086 | 8,327,412,900 | 86.64% |
| JW2372_0 h | Replicate 1 | 59,790,518 | 8,968,577,700 | 84.91% |
| JW2372_0 h | Replicate 2 | 67,703,216 | 10,155,482,400 | 84.66% |
| JW2372_0 h | Replicate 3 | 54,237,882 | 8,135,682,300 | 84.09% |
| JW2372_2 h | Replicate 1 | 55,640,330 | 8,346,049,500 | 83.46% |
| JW2372_2 h | Replicate 2 | 56,135,152 | 8,420,272,800 | 82.99% |
| JW2372_2 h | Replicate 3 | 67,027,818 | 10,054,172,700 | 84.38% |
| JW2372_12 h | Replicate 1 | 51,308,406 | 7,696,260,900 | 83.83% |
| JW2372_12 h | Replicate 2 | 62,567,520 | 9,385,128,000 | 84.55% |
| JW2372_12 h | Replicate 3 | 52,513,498 | 7,877,024,700 | 85.26% |
